# Thunderbeat®: a new step forward in transoral surgery—systematic review of literature and our experience

**DOI:** 10.1007/s00405-023-07944-8

**Published:** 2023-04-04

**Authors:** Carmelo Saraniti, Verro Barbara

**Affiliations:** Department of Biomedicine, Neuroscience and Advanced Diagnostic, ENT Clinic, University of Palermo, Palermo, Italy

**Keywords:** Operative surgical procedures, Surgical instruments, Ultrasonic surgical procedures, Transoral surgery, Head and neck surgery, Head and neck cancer

## Abstract

**Introduction:**

Minimally invasive surgery is today the main challenge of ENT surgeons who aim to achieve oncological radicality with less aesthetic and functional impact. This is the basis for the widespread transoral surgical techniques, as the Thunderbeat^®^.

**Objective:**

To date, the use of Thunderbeat^®^ in transoral surgery is still little known and widespread. So, this study analyzes, with a systematic review, current literature about the transoral use of Thunderbeat^®^ and shows our case studies.

**Methods:**

The research was carried out on Pubmed, Scopus, Web of Science and Cochrane databases using specific keywords. Then, a retrospective study was carried out on 10 patients who underwent transoral surgery by Thunderbeat^®^ in our ENT Clinic. Both in our cases and in the systematic review the following parameters have been evaluated: treated anatomical site and subsite, histological diagnosis, type of surgery, duration of nasogastric tube and hospitalization, post-operative complications, tracheostomy, resection margin status.

**Results:**

The review included 3 articles that described transoral use of Thunderbeat^®^ for a total of 31 patients suffering from oropharyngeal, hypopharyngeal and/or laryngeal carcinoma. Nasogastric tube was removed after 21.5 days on average, temporary tracheostomy was performed in 6 patients. The main complications were: bleeding (12.90%) and pharyngocutaneous fistula (29.03%). Thunderbeat^®^ shaft was 35 cm long and 5 mm large. Our case studies included 5 males and 5 females, mean age 64.4 ± 10.28, with oropharyngeal or supraglottic carcinoma, parapharyngeal pleomorphic adenoma and cavernous hemangioma of the tongue base. Temporary tracheostomy was performed in 8 patients. Free resection margins were achieved in all cases (100%). No peri-operative complications occurred. Nasogastric tube was removed after 5.3 ± 2 days on average. All patients were discharged without tracheal tube and NGT after 18.2 ± 4.72 days on average.

**Conclusion:**

This study demonstrated that Thunderbeat^®^ has several advantages over other transoral surgical approaches, such as CO2 laser and robotic surgery, in terms of best combination of oncological and functional success, less post-operative complications and costs. So, it could represent a step forward in transoral surgery.

## Introduction

Minimally invasive surgery (MIS) is today the main challenge of ENT surgeons who aim to achieve oncological radicality with less aesthetic and functional impact. This is the basis for the spreading of transoral surgical techniques: electrocauterization, transoral laser microsurgery (TOLM), transoral robotic surgery (TORS) and lastly transoral ultrasonic surgery (TOUSS). Compared to classical transcervical or open surgery of the head and neck region, transoral surgery is performed without external incisions ensuring a complete oncological resection of the primary tumor through the mouth.

Different cutting tools can be used during transoral surgery. The ideal surgical tool should allow efficient cutting and hemostasis with minimal thermal damage [1]. As Tirelli et al. stated when choosing the surgical tool, the surgeon must keep in mind its effects of thermal damage on adjacent safe tissue and on histological examination of the resection margins, its coagulation ability, and the healing of the wound [2].

The TOUSS was born with the deployment in transoral surgery of a new generation device that combines ultrasound (US) and radiofrequency (RF)—Thunderbeat^®^ (Olympus Medical Systems Corp., Tokyo, Japan)—first used for laparoscopic, urological, and gynecological surgery. The combination of US and RF in a single device provides, at the same time, efficient sealing of up to 7 mm vessel and fast cutting with minimal thermal damage on closer safe tissue [3]. Thus, Thunderbeat^®^ represents a new intuitive and effective procedure, easily available, and less expensive than TORS. About this, Fernández-Fernández MM et al., the first to introduce this device in transoral surgery, explain that Thunderbeat^®^ achieves the same outcomes as the daVinci surgical system (Intuitive Surgical, Sunnyvale, CA) with low costs [4].

To date, however, the use of Thunderbeat^®^ in transoral surgery is still little known and widespread. For this reason, to study and better know this device that could represent the best combination of oncological and functional success plus careful on costs, this study has two goals: analyzing, with a systematic review of the current literature about the transoral use of Thunderbeat^®^ and showing our case studies, therefore our point of view on the TOUSS, the new step forward of transoral surgery.

## Material and methods

### Systematic review: search methodology

The selection of manuscripts was carried out on the main databases Pubmed, Scopus, Web of Science and Cochrane, using the following keywords: *transoral* AND *thunderbeat* OR *ultrasonic* AND *larynx* OR *oropharynx* OR *head and neck* OR *surgery* (Table 1). Moreover, some articles have been identified in the bibliography of selected studies.

**Table 1:** Search methodology

Databases	Keywords				
Pubmed	*Transoral*	AND	*Thunderbeat* OR *Ultrasonic*	AND	*Larynx* OR *Oropharynx* OR *Head and neck* OR *Surgery*
Scopus					
Web of Science					
Cochrane					

Two independent authors (BV and CS) examined the manuscripts, and a preliminary selection was performed only based on the title and abstract. Subsequently, the selected articles were read entirely to include in the study only those who met the eligibility criteria.

The systematic review was carried out according to the PRISMA guidelines and the protocol was recorded on the PROSPERO system.

### Systematic review: eligibility criteria

The inclusion criteria for articles selected were: (1) prospective and/or retrospective studies about the use of Thunderbeat^®^ in TOUSS; (2) studies providing data about diagnosis, possible post-operative complications, treated anatomical site, resection margins status; (3) studies about benign and/or malignant head and neck diseases.

However, the exclusion criteria were as follows: (1) articles not written in English; (2) reviews, editorials, opinions or thesis; (3) studies that didn’t specify the use of Thunderbeat^®^ during surgery; (4) studies where Thunderbeat^®^ was not used for transoral surgery.

### Our case studies: study protocol

A retrospective study was conducted on patients undergoing transoral surgery by Thunderbeat^®^ due to benign and/or malignant head and neck disease in our ENT clinic from September 2019 to September 2022.

Informed consent to participate in the study was obtained by the patients and the study was conducted in accordance with the Declaration of Helsinki and approved by the ethics committee of our University Hospital (approval number 10/2022).

Inclusion criteria were: (1) females and males; (2) older than 18 years old; (3) head and neck benign and/or malignant lesion with an indication for surgery; (4) informed consent for TOUSS signed by the patient.

Exclusion criteria were: (1) pregnancy; (2) inability to understand the surgical procedure; (3) lack of data in the database about the parameters to analyze; (4) any contraindication to surgery; (5) disease that cannot be surgically removed; (6) refusal of the patient to surgical intervention.

### Data analysis

Both for the articles selected for systematic review and for the patients of our casuistry, the following parameters were analyzed: treated anatomical site and subsite, histological diagnosis of the excised lesion, type of surgery performed, duration of a naso-gastric tube (NGT) and hospitalization, any post-operative complications, possible tracheostomy, the effectiveness of surgery in terms of radicality and surgical outcomes.

Duration of NGT and hospitalization were expressed in days. About oncological radicality, resection margins were evaluated: negative (R0), close (R1), positive (R2), not-evaluable (Rx) [5]. Data were collected on spreadsheets using Microsoft Excel (16.66.1 version). Data were reported as numbers, percentages of total and/or mean ± standard deviation (SD).

## Results

**Fig. 1:**
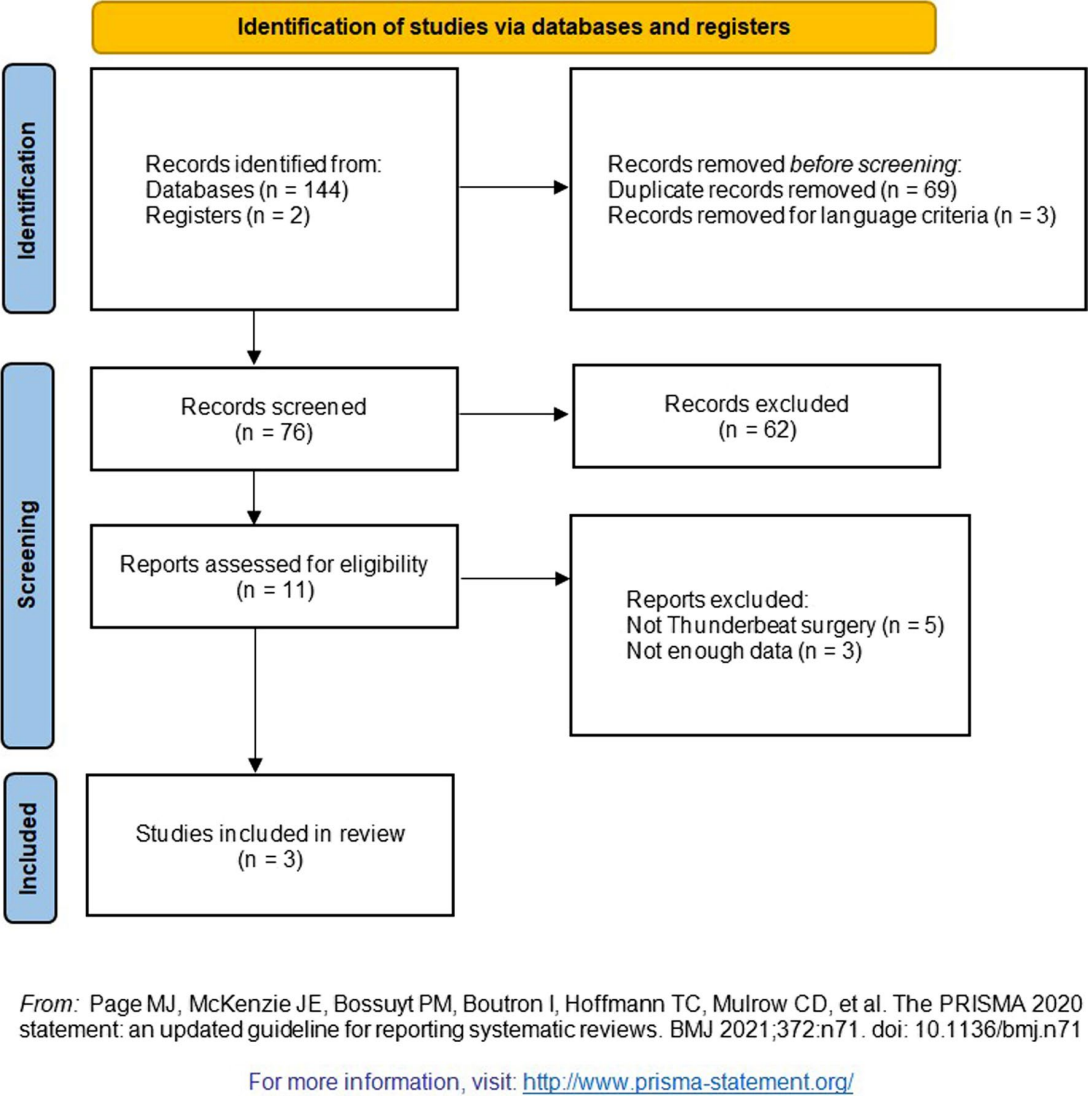
PRISMA 2020 Flow Diagram © of the study selection process of literature

Figure 1 shows the selection process of studies according to Preferred Reporting Items for Systematic Reviews and Meta-Analysis (PRISMA)© 2020 flow diagram [6]. Overall, from systematic research on databases and bibliography 145 articles have been selected. So, after eliminating the duplicates (no. 69), 76 articles were evaluated and of these 65 were excluded by reading title and abstract because not consistent with the topic of research or because not written in English. Finally, the two authors (BV and CS) independently read the full text of 11 articles and based on selection criteria, included only 3 manuscripts in the present review (Table 2).

**Table 2:** Characteristics of included studies in the systematic review

Authors (year of publication)	Fernández-Fernández MM et al. (2015) [7]	Fernández-Fernández MM et al. (2016) [4]	Sakthivel et al. (2022) [3]
Type of study	Prospective	Not specified	Prospective
No. patients	11	2	18
Treated anatomical site	Oropharynx (6) Hypopharynx (2) Oro-hypopharynx (1) Supraglottis (2)	Larynx (2)	Oral cavity (2) Oropharynx (13) Supraglottis (3)
Disease	Cancer	Cancer	Cancer
Type of surgery	TOUSS primary tumor removal + unilateral or bilateral neck dissection (8) + tracheostomy (4)	TOUSS total laryngectomy	TOUSS primary tumor removal + unilateral or bilateral neck dissection (14)
Resection margins	Negative (10) Not-evaluable (1)	Negative (2)	Negative (10) Close (5) Positive (3)
Duration of NGT (days)	Average 10.4 (range 0–28)	Average 37.5 (range 13–49)	Average 16.6 (range 3–22)
Duration of hospitalization (days)	Average 14.3 (range 1–43)	Not specified	Average 7.4 (range 2–16)
Post-operative complications	Bleeding (2) PCF (4) Internal jugular vein thrombosis (1)	PCF (1)	Bleeding (2) Tracheostomy (2) PCF (4)
Tracheostomy (no. patients)	4	2	2
Thunderbeat^®^ shaft	Length: 35 cm Diameter: 5 mm	Length: 35 cm Diameter: 5 mm	Length: 35 cm Diameter: 5 mm

TOUSS TransOral UltraSonic Surgery, NGT naso-gastric tube, PCF pharyngocutaneous fistula

In particular, the article by Fernández-Fernández et al. [7] is the first study about TOUSS as an alternative to TORS in head and neck disease. In this study, they reported 11 cases of patients with cancer of the following anatomical sites: oropharynx (no. 6), hypopharynx (no. 2), oro-hypopharynx (no. 1) and supraglottis (no. 2). In one case, both Thunderbeat^®^ and CO2 laser were performed due to glottic extention of cancer. Oncological radicality was achieved in all patients with 10 cases of free resection margins (R0) and 1 case of not-evaluable resection margin (Rx). Prophylactic tracheostomy was performed during surgery in 4 patients. Complications occurred in a small percentage of patients: post-operative bleeding (no. 2), pharyngo-cutaneous fistula (PCF) (no. 4) and internal jugular vein thrombosis (no. 1). This last complication, however, is not to be ascribed to the surgical procedure but to the advanced stage of the tumor as the patient already had metastases. NGT was placed in 8 patients and removed after 10.4 days on average (range 0–28 days); the patient with metastases underwent gastrostomy. Authors point out that patients were discharged only after the removal of NGT and closure of tracheostomy.

Another study included in this systematic review reports the results of two TOUSS total laryngectomy due to laryngeal carcinoma performed by Thunderbeat^®^ [4]. Resection margins resulted negative in both cases. In one case a pharyngo-cutaneous fistula has developed, probably due to previous radiotherapy. NGT was removed after 31 days on average (range 13–49 days). The study does not report the duration of hospitalization although it is likely that patients were discharged after NGT removal and closure of PCF.

The last study reports 18 cases of carcinoma of the following anatomical sites: oral cavity (no. 2), oropharynx (no. 13) and supraglottis (no. 3) [3]. In the 3 cases of supraglottic cancer, the use of Thunderbeat^®^ was combined with a CO2 laser to ensure oncological radicality close to the glottis. In 10 cases (55.55%) resection margins were negative, in 5 cases (27.78%) they were close (1–5 mm) and in 3 cases (16.67%) they were positive (> 5 mm). All 3 positive margins were obtained in oropharyngeal surgery. In 2 cases (oropharynx) there was a post-operative bleeding that needed a temporary tracheostomy. More frequent was the development of PCF that occurred in 5 patients (27.78%). NGT was placed in all patients and removed after 16.6 days on average (range 2–16 days). Some patients were discharged with the NGT still in place.

In all three studies included in the systematic review, the same type of Thunderbeat^®^ (shaft length 35 cm and diameter 5 mm) was used regardless of the anatomical site.

### Our case studies

According to the selection criteria, 10 patients were included in the study: 5 males and 5 females, aged between 49 and 81 years of age (mean age 64.4 ± 10.28). The involved anatomical sites were: oropharynx (no. 6), supraglottis (2) and parapharyngeal space (2). In particular, 2 patients had supraglottic carcinoma, 2 patients had deep lobe parotid gland (parapharyngeal spreading) pleomorphic adenoma, 1 patient had a cavernous hemangioma of the tongue base, 2 patients had soft palate carcinoma and 2 retromolar trigone carcinoma. Temporary tracheostomy was performed in 8 patients (80%) treated for oropharyngeal disease: in one case (case #1), tracheostomy was performed due to difficult intubation rather than the surgical risk. In case #3, where the supraglottic carcinoma extended to the anterior commissure, the use of Thunderbeat^®^ was combined with a CO2 laser. After supraglottic surgery, the surgical field doesn’t need reconstruction, since it heals by secondary intention. In parapharyngeal pleomorphic adenoma, we performed a direct wound closure. For what concerns oropharyngeal cancer, no reconstruction procedure was performed. None of the patients with soft palate excision suffered from velopharyngeal incompetence with nasal regurgitation. Only two patients (cases #6 and #8) developed post-operative rhinolalia aperta: case #6 had moderate degree of rhinolalia so he didn’t need plastic surgery, case #8 had severe rhinolalia and he’s candidate for surgery for velopharyngeal insufficiency. However, during follow-up, this latter patient developed a hepatocarcinoma, so he’s on treatment for this second tumor.

**Fig. 2:**
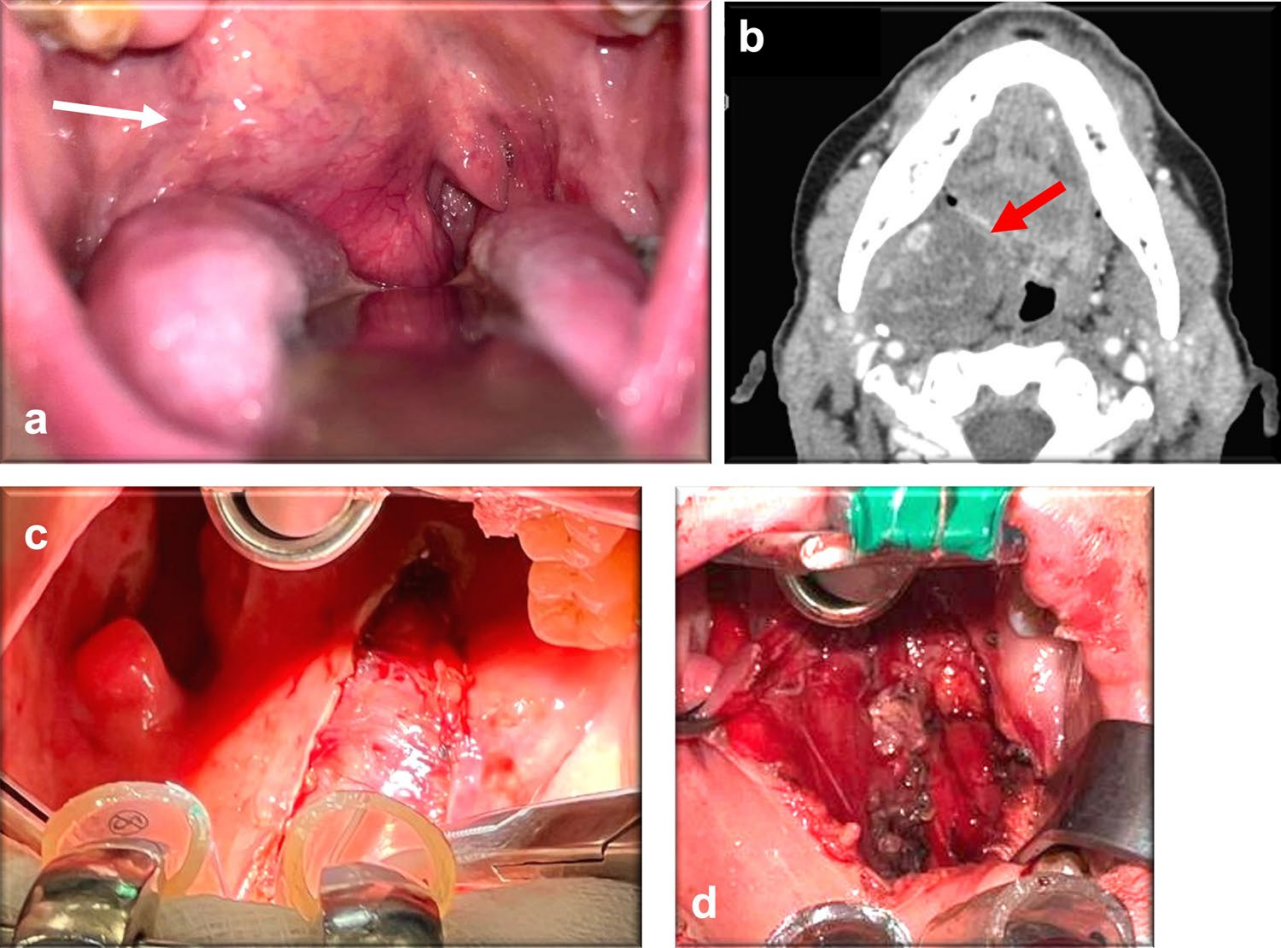
Case #1: Right deep lobe parotid gland (parapharyngeal spreading) pleomorphic adenoma on oropharyngoscopy (**a**) and axial computed tomography (**b**), intra-operative images pre (**c**) and after (**d**) removal of lesion

**Fig. 3:**
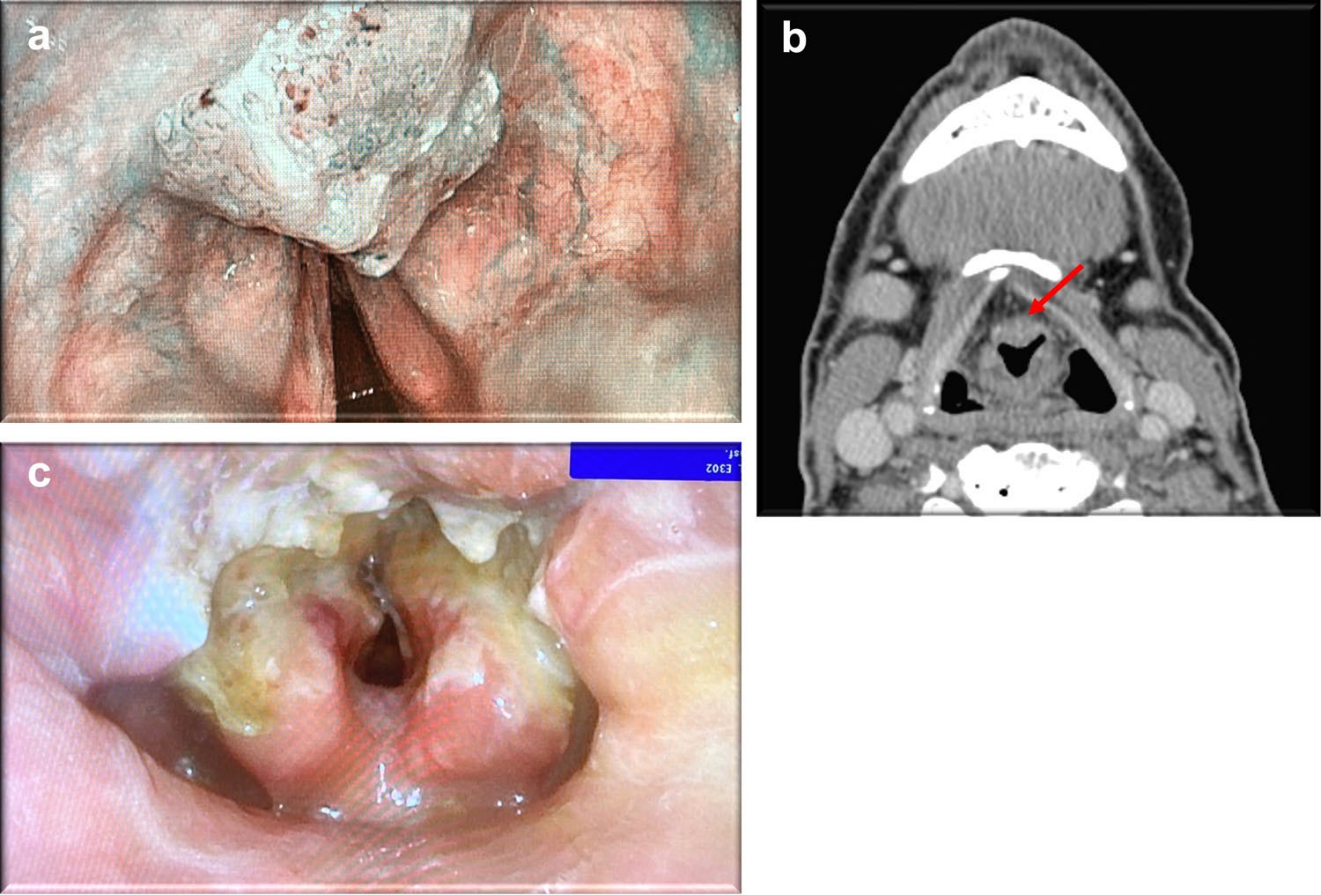
Case #2: Lesion of the laryngeal surface of epiglottis on narrow-band imaging (NBI) laryngoscopy (**a**) and axial computed tomography showing free pre epiglottic space (red arrow) (**b**), laryngoscopic image one day after surgery (**c**)

**Fig. 4:**
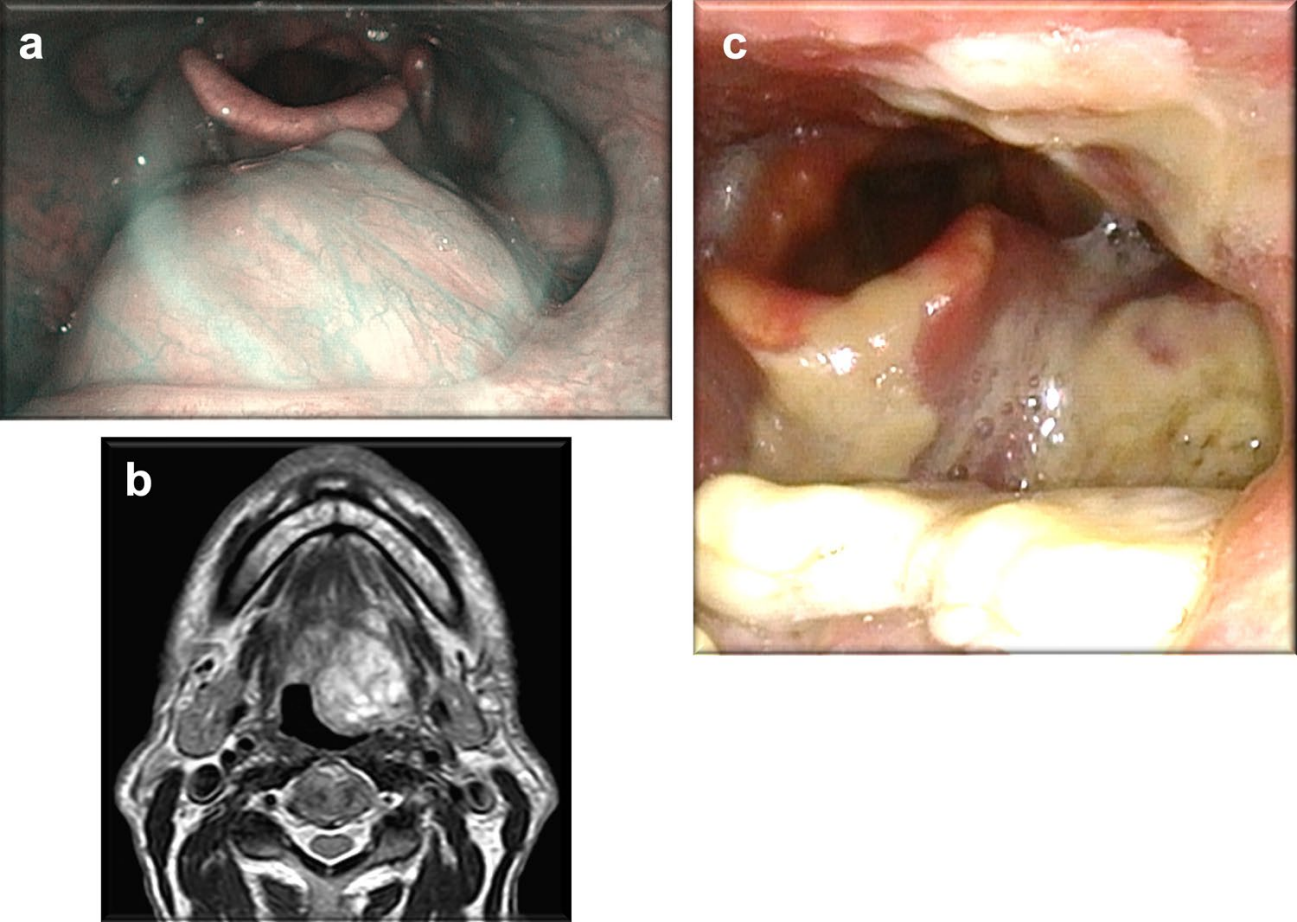
Case #4: Submucosal lesion on the left of base tongue, with a rich vascular network on covering mucosa on NBI laryngoscopy (**a**) and T2 scan axial magnetic resonance showing oval lesion with regular margins and homogeneous contrast medium (**b**), laryngoscopic image one week after surgery (**c**)

**Fig. 5:**
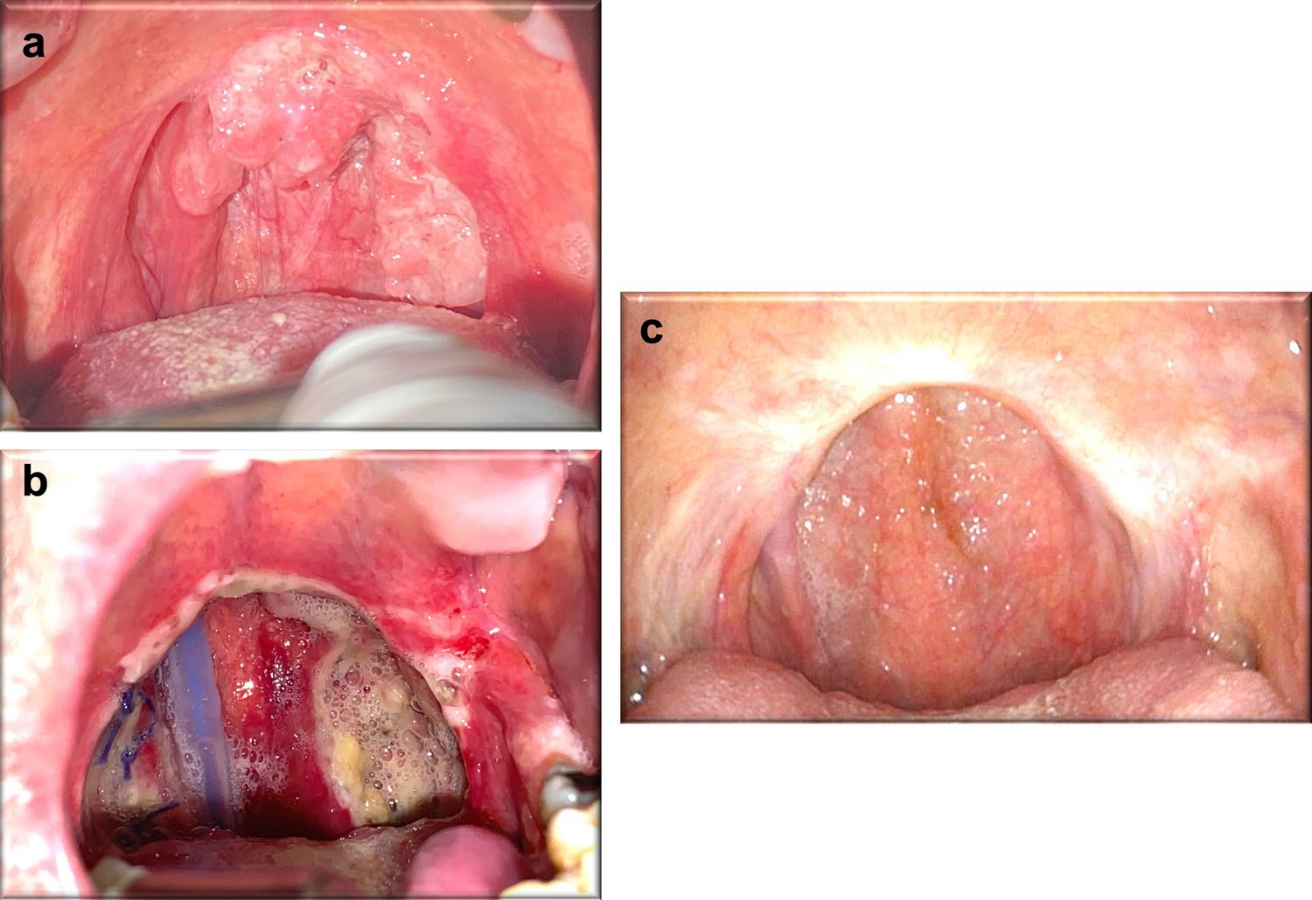
Case #8: squamous cell carcinoma of the soft palate and left palatin tonsil (**a**), 5° post-operative day with nasogastric tube (**b**), 3° post-operative month (**c**)

The features of Thunderbeat^®^ were different according to the anatomical site treated: its shaft length was 35 cm for supraglottic and tongue base lesions and 20 cm for closer oropharyngeal lesions. Free resection margins were achieved in all cases (100%). No peri-operative complications occurred, and no patients needed blood transfusions. NGT was removed after 5.3 ± 2 days on average (range 1 – 7). All patients were discharged without tracheal tube and NGT. Hospitalization lasted 18.2 ± 4.72 days on average (range 12–26) (Tables 3, 4) (Figs. 2, 3, 4, 5).

**Table 3:** Characteristics of our case studies

Characteristics	N (%)
**Sex**	
Male	5 (50)
Female	5 (50)
**Age (years)**	
Mean ± SD	64.4 ± 10.28
Range	49 – 81
**Treated anatomical site**	
Oropharynx	6 (60)
Parapharyngeal space	2 (20)
Supraglottis	2 (20)
**Disease**	
SCC	7 (70)
Pleomorphic adenoma	2 (20)
Cavernous hemangioma	1 (10)
**Type of surgery**	
Wide local excision (soft palate, tonsils, retromolar trigone)	5 (50)
Tumor removal (pleomorphic adenoma)	2 (20)
Partial glossectomy	1 (10)
ESL type IIIa	1 (10)
ESL type IIIb + CO2 laser	1 (10)
**Resection margins**	
Free	10 (100)
Close	0
Positive	0
Not-evaluable	0
**Duration of NGT (days)**	
Mean ± SD	5.3 ± 2
Range	1–7
**Duration of hospitalization (days)**	
Mean ± SD	18.2 ± 4.72
Range	12 – 26
Post-operative complications	
Bleeding	0
Pharyngo-cutaneous fistula	0
Others	0
**Tracheostomy**	
Yes	8 (80)
Permanent tracheostomy	0
Not	2 (20)
**Thunderbeat**^®^ **shaft**	
***Length***	
Tongue base surgery	35 cm
Soft palate, retromolar trigone, tonsil surgery	20 cm
Parapharyngeal space surgery	20 cm
Supraglottic surgery	35 cm
*** Diameter***	5 mm
**Total**	10 (100)

SD standard deviation, SCC squamous cell carcinoma, ESL endoscopic supraglottic laryngectomy [8]

**Table 4:** Our case series

Case	Sex	Age (years)	Treated anatomical site	Disease	Type of surgery	Tracheostomy	Resection margins	Duration of NGT (days)	Duration of hospitalization (days)	Post-op compl	Thunderbeat^®^ shaft
Case #1	F	81	Parapharyngeal space	Right deep lobe parotid gland (parapharyngeal spreading) pleomorphic adenoma	TOUSS tumor removal + tracheostomy	Yes	R0	5	15	None	Length: 20 cm Diameter: 5 mm
Case #2	M	58	Supraglottis	Squamous cell carcinoma pT1*	TOUSS ESL type IIIa	None	R0	1	20	None	Length: 35 cm Diameter: 5 mm
Case #3	F	78	Supraglottis—glottis	Squamous cell carcinoma pT3*	TOUSS ESL type IIIb + CO2 laser extension to anterior commissure	None	R0	3	13	None	Length: 35 cm Diameter: 5 mm
Case #4	M	72	Oropharynx	Cavernous hemangioma	TOUSS partial glossectomy + tracheostomy	Yes	R0	4	12	None	Length: 35 cm Diameter: 5 mm
Case #5	F	64	Parapharyngeal space	Right deep lobe parotid gland (parapharyngeal spreading) pleomorphic adenoma	TOUSS tumor removal + tracheostomy	Yes	R0	5	14	None	Length: 20 cm Diameter: 5 mm
Case #6	M	67	Oropharynx	Squamous cell carcinoma pT2*	TOUSS partial glossectomy + wide local excision (left tonsil, tonsil pillars, retromolar trigone) + left neck dissection + tracheostomy	Yes	R0	7	26	None	Length: 20 cm Diameter: 5 mm
Case #7	M	49	Oropharynx	Squamous cell carcinoma pT2*	TOUSS wide local excision (soft palate, left tonsil) + tracheostomy	Yes	R0	7	21	None	Length: 20 cm Diameter: 5 mm
Case #8	M	52	Oropharynx	Squamous cell carcinoma pT2*	TOUSS wide local excision (soft palate, both tonsils) + tracheostomy	Yes	R0	7	24	None	Length: 20 cm Diameter: 5 mm
Case #9	F	55	Oropharynx	Squamous cell carcinoma pT2*	TOUSS wide local excision (left tonsil, tonsil pillars, retromolar trigone) + tracheostomy	Yes	R0	7	15	None	Length: 20 cm Diameter: 5 mm
Case #10	F	68	Oropharynx	Squamous cell carcinoma pT2*	TOUSS wide local excision (right soft hemypalate, tonsil, tonsil pillars, retromolar trigone) + right neck dissection + tracheostomy	Yes	R0	7	22	None	Length: 20 cm Diameter: 5 mm

NGT naso-gastric tube, TOUSS TransOral UltraSonic Surgery, ESL endoscopic supraglottic laryngectomy [8], post-op compl post-operative complication

*According to the 8th edition AJCC [13]

## Discussion

Over the last decade, the trend to minimally invasive surgery (MIS) has led to new technologies, as Thunderbeat^®^, in the surgical armamentarium of head and neck disease. Indeed, until about 10 years ago, in the head and neck region, Thunderbeat^®^ was used only in case of open surgery (e.g., thyroidectomy, parotidectomy).

Fernandez-Fernandez et al. were the first to perform a TOUSS using the Thunderbeat^®^ device both for oropharyngeal and supraglottic cancers [7]. With their study, they demonstrated the effectiveness of this device that combines two energies: ultrasound and radiofrequency. US ensures fast and precise tissue cutting by denaturing proteins and breaking hydrogen bonds at low temperature, while RF provides efficient sealing of up to 7 mm vessel: the combination of these energies allows the surgeon to perform faster, safer and more precise procedures, without the need to change the surgical instrument in case of bleeding. on the contrary, CO2 laser can coagulate less than 0.5 mm vessels [9] and so, neither TOLM nor TORS can tackle bleeding from larger vessels (5–7 mm in diameters) [10]. Indeed, as we know, bleeding is the main complication of surgery, due to both the treated anatomical site and transoral approach that does not include vessel ligation. Thunderbeat^®^ ability to seal vessels up to 7 mm in diameter allows the surgeon to perform surgery safely at oropharyngeal and supraglottic levels where he may encounter lingual or superior laryngeal arteries. This advantage is confirmed by current literature as well as by our case studies. Indeed, post-operative bleeding occurred only in 4 out of 31 cases (12.90%) [3, 7] of the systematic review and no one occurred in our cases (0/10).

Another complication found in the systematic review was PCF that occurred in 29.03% of cases. The PCF is another complication to correlate both to the anatomical site and to the surgical procedure, regardless of the approach (transoral or open). Indeed, external surgical techniques are associated with an increased risk of developing PCF [11]. In their review, Mäkitie et al. found that the incidence of PCF after pharyngo-laryngeal surgery is between 9 and 23% and have identified the following risk factors: comorbidities, malnutrition, low hemoglobin levels, previous radiotherapy, tumor stage, an extension of resection, duration of NGT [12]. Actually, in their study, Fernández-Fernández et al. justified the development of pharyngo-cutaneous fistula after total laryngectomy due to previous radiotherapy [4]; while in the 2015 study, the authors stated that PCF should not be considered a complication, but a mandatory condition given the extent of surgical resection [7], highlighting the role of radiotherapy in development of PCF, without specifying if neck dissection was performed during the same procedure. Sakthivel et al. reported 4/18 cases of PCF in patients undergoing concurrent neck dissection: three of them had intraoperative cervico-pharyngeal communication due to extended resection. Moreover, all four patients were treatment naïve [3]. So, in this study, PCF seems to be related to tumor resection and concurrent neck dissection. In our casuistry, no cases of PCF have been reported although we have treated oropharyngeal, parapharyngeal and supraglottic lesions and despite some patients had a few of above-mentioned risk factors. Indeed, two patients suffered from type 2 diabetes mellitus, 5 patients had pT2 oropharyngeal squamous cell carcinoma (SCC) and 1 patient had pT3 supraglottic SCC (according to 8^th^ edition of the American Joint Committee on Cancer) [13].

NGT was placed in all patients with two purposes: for prophylactic aim, that is to avoid injuries and therefore bleeding of the operated site, and second to avoid possible post-operative dysphagia. In our case studies, where the risk of bleeding was lower, therefore in endoscopic supraglottic laryngectomies, NGT was removed 1–3 days on average after surgery; in the case of oropharyngeal and parapharyngeal surgeries, NGT was removed between 4 and 7th post-operative day. Overall, in our casuistry, NGT was removed after 5.3 ± 2 days on average after surgery. On the contrary, in their studies, Fernández-Fernández et al.[7] and Sakthivel et al. [3] reported that their patients have been fed with NGT for 10.4 days and 16.6 days on average, respectively.

As written above, oncological radicality and organ function preservation represent the two main goals of conservative surgery. So, free resection margins and minimal damage to adjacent tissues must be ensured. The systematic review revealed that resection margins were negative in 22 cases (70.97%), positives in 3 cases (9.68%), close in 5 cases (16.13%) and not-evaluable in 1 case (3.22%). In our case studies, 100% of cases had free resection margins, both in oropharyngeal and laryngeal diseases. However, it’s important to point out that this type of surgical devices causes a thermal tissue injury that may impair the assessment of the resection margins due to carbonization and shrinkage [14, 15]. Mandelli et al. compared CO2 laser, harmonic scalpel (that uses US energy) and monopolar electrocautery and their analysis revealed that monopolar electrocautery causes the highest thermal tissue injury, and that harmonic scalpel causes the highest tissue retraction [14]. Another study compared three TOUSS devices: LigaSure^®^ (Valleylab Inc., Boulder, CO, USA) that use RF energy, Harmonic ACE^®^ (Ethicon Endo-Surgery, Cincinnati, OH, USA) that uses US energy and Thunderbeat^®^ that combines US and RF energies. The study reported that Thunderbeat^®^ has a capacity of coagulating vessels between 5 and 7 mm in diameter slightly better than LigaSure® and significantly better than Harmonic ACE^®^ [16]. Moreover, Thunderbeat^®^ has ensured faster dissection and cutting and lower lateral thermal damage [17, 18]. Low adjacent tissue injury leads to rapid healing of the surgical site and shorter hospitalization. These results are confirmed by the quick recovery of our patients that have been discharged 18.2 ± 4.72 days on average after surgery as well as reported by studies included in the systematic review.

As well as for all transoral surgery, also TOUSS has two main critical points: the inside-out anatomy and the proper exposure. Surgeons usually study anatomy from the outside to the inside; on the contrary, approaching transoral surgery, regardless of the instrument, surgeons have to change perspective and approach to a reversed anatomy. Hinni et al. have drawn up a list of 10 factors (the so-called “10 T”) influencing exposure: teeth, tongue, tilt (neck extension), tori, trismus, tumor, previous treatment, transverse diameter of the mandible, tethering (fibrosis) and compression time [19]. DaVinci robot can’t be used in case of reduced mouth opening due to its arms dimensions. On the contrary, Thunderbeat^®^ can be used also in case of limited mouth opening. Indeed, in our case (case #1) of difficult intubation due to reduced neck extension and reduced mouth opening, a tracheostomy was performed to induce general anesthesia. However, Thunderbeat^®^ was used to excise deep lobe parotid gland (with parapharyngeal spreading) pleomorphic adenoma although on patients with limitations. From the systematic review as well as from our case studies an important data has emerged: the difficulty of ensuring oncological radicality by Thunderbeat^®^ in the case of tumor that reaches the glottic level (anterior commissure). In fact, the jaws of Thunderbeat^®^ are still too bulky to work close to the vocal cords. Because of this, when supraglottic cancer extended to the anterior commissure, we used both Thunderbeat^®^ and CO2 laser as Fernández-Fernández et al. [7]and Sakthivel et al. [3] also performed in these cases. However, this possibility of combining the two surgical techniques could be considered also an advantage of Thunderbeat^®^.

This study presents a major limit that also represents its strength: the small number of studies, and, therefore, of clinical cases, about the use of Thunderbeat^®^ in transoral surgery. Equally small is to date our case studies although it encompasses different diseases, anatomical sites, and surgeries.

## Conclusions

Today, surgery aims to be less invasive and more conservative in compliance with the principles of oncological radicality. On the assumption of MIS, transoral surgeries were born: TOLM, TORS and lastly TOUSS. Each surgical technique has advantages and limitations that Thunderbeat^®^ tries to integrate and overcome: simultaneous ability to seal and cut with lower risk of perioperative bleeding, faster preparation and execution, lower cost, faster learning curve. However, further studies with larger samples are necessary to provide a statistically significant result to what is clear and evident in clinical experience: Thunderbeat^®^ is the—almost—perfect combination between TORS and TOLM, a new step forward in transoral surgery.
