# Correction: Thunderbeat^®^: a new step forward in transoral surgery—systematic review of literature and our experience

**DOI:** 10.1007/s00405-023-07993-z

**Published:** 2023-04-25

**Authors:** Carmelo Saraniti, Barbara Verro

**Affiliations:** Department of Biomedicine, Neuroscience and Advanced Diagnostic, ENT Clinic, University of Palermo, Palermo, Italy

**Correction: European Archives of Oto-Rhino-Laryngology** 10.1007/s00405-023-07944-8

In the original publication of the article, second author’s name was published incorrectly as “Verro Barbara”. The correct name should read as follows “Barbara Verro”.

